# Kidins220/ARMS modulates brain morphology and anxiety-like traits in adult mice

**DOI:** 10.1038/s41420-022-00854-4

**Published:** 2022-02-09

**Authors:** Amanda Almacellas-Barbanoj, Martina Albini, Annyesha Satapathy, Fanny Jaudon, Caterina Michetti, Alicja Krawczun-Rygmaczewska, Huiping Huang, Francesca Manago, Francesco Papaleo, Fabio Benfenati, Fabrizia Cesca

**Affiliations:** 1 Center for Synaptic Neuroscience and Technology, Fondazione Istituto Italiano di Tecnologia, 16132 Genova, Italy; 2 Department of Experimental Medicine, University of Genova, 16132 Genova, Italy; 3 Department of Life Sciences, University of Trieste, 34127 Trieste, Italy; 4 Genetics of Cognition Laboratory, Neuroscience area, Istituto Italiano di Tecnologia, via Morego, 30, 16163 Genova, Italy; 5 IRCCS Ospedale Policlinico San Martino, 16132 Genova, Italy; 6 Present Address: Clinical and Experimental Epilepsy, UCL Queen Square Institute of Neurology, London, UK

**Keywords:** Neuroscience, Neurotrophic factors

## Abstract

Kinase D interacting substrate of 220 kDa (Kidins220), also known as ankyrin repeat-rich membrane spanning (ARMS), is a transmembrane scaffold protein that participates in fundamental aspects of neuronal physiology including cell survival, differentiation, and synaptic plasticity. The Kidins220 constitutive knockout line displays developmental defects in the nervous and cardiovascular systems that lead to embryonic lethality, which has so far precluded the study of this protein in the adult. Moreover, Kidins220 mRNA is tightly regulated by alternative splicing, whose impact on nervous system physiology has not yet been addressed in vivo. Here, we have asked to what extent the absence of Kidins220 splicing and the selective knockout of Kidins220 impact on adult brain homeostasis. To answer this question, we used a floxed line that expresses only the full-length, non-spliced Kidins220 mRNA, and a forebrain-specific, CaMKII-Cre driven Kidins220 conditional knockout (cKO) line. Kidins220 cKO brains are characterized by enlarged ventricles in the absence of cell death, and by deficient dendritic arborization in several cortical regions. The deletion of Kidins220 leads to behavioral changes, such as reduced anxiety-like traits linked to alterations in TrkB-BDNF signaling and sex-dependent alterations of hippocampal-dependent spatial memory. Kidins220 floxed mice present similarly enlarged brain ventricles and increased associative memory. Thus, both the absolute levels of Kidins220 expression and its splicing pattern are required for the correct brain development and related expression of behavioral phenotypes. These findings are relevant in light of the increasing evidence linking mutations in the human KIDINS220 gene to the onset of severe neurodevelopmental disorders.

## Introduction

Neurodevelopmental disorders (NDDs) disturb central nervous system development and affect more than 3% children worldwide [1]. Several hundred genes are associated to NDDs, converging on pathways for neuronal migration, synaptic plasticity, gene expression, and protein synthesis. The biological underpinnings of NDDs are rarely understood [1] and accordingly, there are no resolutive treatments [2]. Amongst genetic NDDs, we study the Spastic paraplegia, Intellectual disability, Nystagmus and Obesity (SINO) syndrome [3], whose symptoms include motor impairments, mental retardation, defects in vision, obesity, dilated brain ventricles, and a moderate developmental delay. Genetic analysis identified heterozygous de novo nonsense mutations in the KIDINS220 gene. Homozygous KIDINS220 mutations lead to spontaneous abortion [4, 5], while heterozygous patients display a spectrum of the above-mentioned symptoms [6–10].

Kinase D interacting substrate of 220 kDa (Kidins220), also known as ankyrin repeat-rich membrane spanning (ARMS), is a transmembrane protein with pleiotropic functions in nervous system development [11, 12]. Kidins220 interacts with the neurotrophin receptors tropomyosin-related tyrosine kinase (Trk) A, B, and C and p75 (p75^NTR^), mediating the signaling triggered by neurotrophins like brain-derived neurotrophic factor (BDNF) [13, 14]. Kidins220 modulates neuronal cytoskeleton during differentiation [15, 16], synaptic plasticity and neuronal excitability [17–20]. Moreover, it contributes to Ca^2+^ and BDNF signaling in astroglia by affecting the store-operated Ca^2+^ entry (SOCE) mechanism, the expression of the transient receptor potential vanilloid-type 4 (TRPV4) channel, the expression and phosphorylation of TrkB and associated pathways [21, 22]. The constitutive ablation of Kidins220 is lethal, causing developmental defects in the nervous and cardiovascular systems [18, 23]. Downregulation of Kidins220 in the mouse brain leads to impaired dendrite branching [24], decreased spine turnover [25], and increased BDNF secretion [26].

Several Kidins220 isoforms are expressed [27]; of note, the Kidins220 isoform pattern has been studied in tissues and various types of primary neurons, but it is still not described in glial populations. BDNF and NGF modulate Kidins220 splicing in primary neurons, and specific protein isoforms have different intracellular localization [27]. On this basis, the Kidins220 floxed line, originally considered equivalent to wild type, has to be reconsidered. In this line, Kidins220 is expressed from the full-length mouse cDNA, which lacks the possibility of splicing. Albeit Kidins220 floxed animals develop normally [18, 23], they express reduced levels of the protein and display ventriculomegaly [28].

In this work we addressed the physiological function of Kidins220 in the adult brain by studying Kidins220^lox/lox^ mice, expressing only the full-length Kidins220 isoform, and CaMKII-Cre-driven conditional knockout (cKO) mice, which lack Kidins220 in forebrain excitatory neurons from the second postnatal week. Behavioral tests showed increased associative memory in Kidins220^lox/lox^ mice, while reduced anxiety-like levels and sex-dependent alterations of hippocampal-dependent spatial memory were specific for cKO mice. Histochemical and biochemical analyses identified altered cortical and hippocampal neuron development and deficits in BDNF-TrkB signaling; anxiety-like levels were partially rescued by chronic administration of the BDNF mimetic 7,8-DHF to cKO mice. Altogether, our data provide the first comparative analysis of the phenotypes induced by the absence of Kidins220 and of its isoforms, laying the basis for understanding the pathogenic mechanisms underlying SINO and related pathologies.

## Results

### Generation of animals bearing the forebrain-specific deletion of Kidins220

**Fig. 1:**
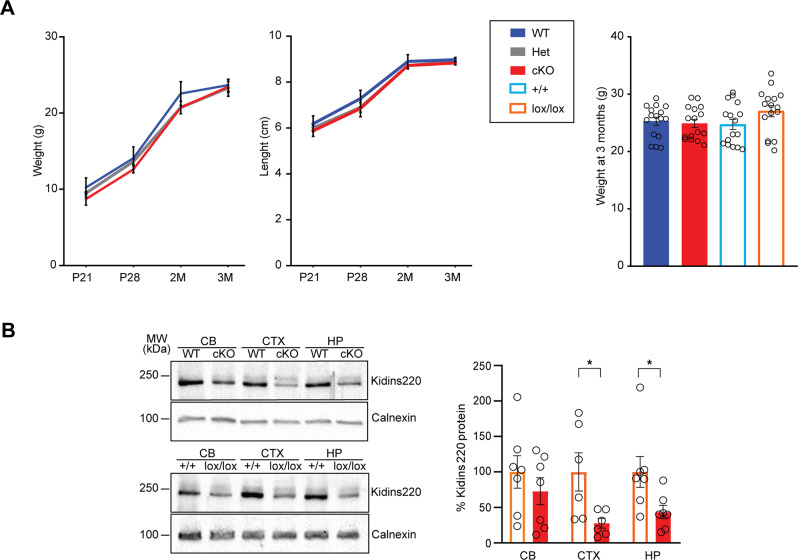
Generation of animals bearing the CaMKII-driven, forebrain-specific deletion of Kidins220. **A** Left: Kidins220^lox/lox;Cre/+^ (cKO, red), Kidins220^lox/+;Cre/+^ (Het, gray), and Kidins220^+/+;Cre/+^ (WT, blue) animals display comparable development for weight (left) and length (middle) up to 3M of age (*n*: P21: 5 WT, 3 cKO, 14 Het; P28: 4 WT, 3 cKO, 7 Het; 2M: 7 WT, 9 cKO, 7 Het; 3M: 16 WT, 13 cKO, 11 Het). Right: Kidins220^+/+^ (+/+, light blue) and Kidins220^lox/lox^ (lox/lox, orange) animals display comparable weight to WT and cKO at 3M of age. One-way ANOVA (*p* > 0.05, *n* = 16 for all the genotypes). **B** Animals were killed at 3M and brains dissected into cerebellum (cb), cortex (ctx), and hippocampus (hp), lysed and analysed by western blotting with anti-Kidins220 antibodies and anti-calnexin antibodies as loading control. Left: representative immunoblots for WT and cKO animals (top), +/+ and lox/lox animals (bottom) are shown. Right: The comparison of Kidins220 expression levels in lox/lox and cKO brain lysates ran on the same nitrocellulose membrane shows a significant reduction of Kidins220 protein in the cortex and hippocampus of cKO mice compared to lox/lox, while in the cerebellum expression levels are comparable. Values are plotted as mean ± S.E.M. and individual values are represented with circles. Unpaired Student’s *t*-test, *p < 0.05 (*n*: Cerebellum: lox/lox 7, cKO 7; Cortex: lox/lox 6, cKO 6; Hippocampus: lox/lox 7, cKO 7).

The postnatal development of cKO, Het, and WT animals, as well as of Kidins220^lox/lox^ [18] and +/+ mice, was comparable up to 3 months (M) of age (Fig. 1A). All genotypes were born at the expected mendelian ratios. Throughout the paper, the four experimental groups are: cKO (Kidins220^lox/lox;+/Cre^) and WT (Kidins220^+/+;+/Cre^); Kidins220^lox/lox^ and +/+ (Kidins220^+/+^).

Kidins220 expression was evaluated in cKO and Kidins220^lox/lox^ brain at 3M. Kidins220 expression was comparable in the two control groups (Fig. S1A); Kidins220^lox/lox^ mice showed about 50% Kidins220 reduction compared to +/+ (Fig. S1B). Kidins220 expression was further reduced in the cKO cortex and hippocampus while in the cerebellum, where the Cre transgene is not expressed, protein levels were comparable (Fig. 1B). A complete ablation of the protein is not expected, since the activation of the CaMKII promoter is restricted to excitatory neurons, leaving Kidins220 expression unaffected in inhibitory neurons and glial cells [19, 21]. The specificity of Cre expression was independently verified by crossing CaMKII-Cre mice with td-tomato^lox/lox^ animals (Fig. S2). Altogether, these data show that: (i) the forebrain-specific knockout of Kidins220 was successfully achieved and (ii) the alteration of Kidins220 splicing pattern causes a global reduction of its expression.

### Kidins220 cKO mice display enlarged brain ventricles in the absence of cell death

**Fig. 2:**
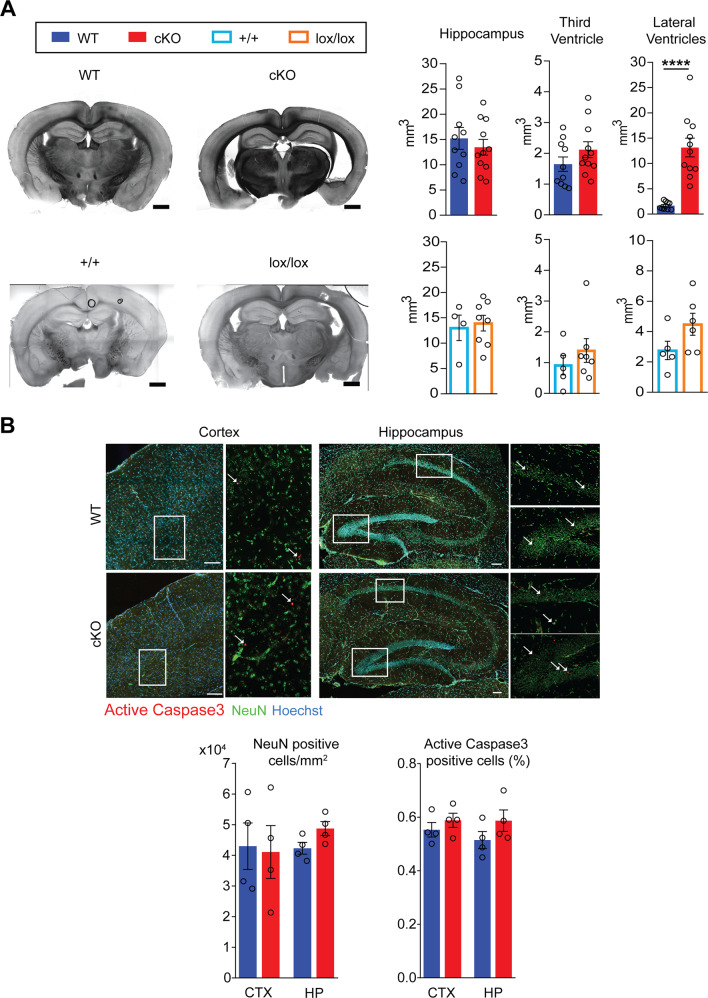
Kidins220 cKO mice display enlarged brain ventricles in the absence of cell death. **A** General brain anatomy was analysed on coronal sections of Kidins220 cKO (red bars), WT (blue), +/+ (light blue), and lox/lox (orange) animals between 2M and 3M of age. Left panel: representative sections from WT, cKO, lox/lox, and +/+ animals are shown. Scale bars: 1 mm. Right panel: quantification of hippocampal volume, third and lateral ventricle volume for WT and cKO (upper) and +/+ and lox/lox animals (lower). Lateral ventricles are enlarged in Kidins220 cKO animals, while all the other parameters did not show statistically significant differences (Student’s *t*-test, ****p < 0.0001; *n*: hippocampus: WT 10, cKO 11, +/+ 4, lox/lox 8; third ventricle: WT 10, cKO 11, +/+ 5, lox/lox 7; lateral ventricle: WT 10, cKO 11, +/+ 5, lox/lox 6). **B** Sagittal sections from cKO and WT mice between 2M and 3M of age were stained with anti-active caspase 3 and anti-NeuN antibodies. Top: Representative images of staining in the hippocampus and somatosensory cortex. Scale bars: 200 μm. Bottom: quantification of neuronal cell density and percentage of active caspase-3 cells (unpaired Student’s *t*-test, *p* > 0.05, *n* = 4 animals per genotype from four separate litters). All data are expressed as means ± S.E.M. and individual values are represented with circles.

Reconstruction of serial coronal sections revealed a significant enlargement of cKO lateral ventricles, while the third ventricle and hippocampal volume were not affected (Fig. 2A, top panel). A similar trend was evident in Kidins220^lox/lox^ mice (Fig. 2A, bottom panel). Neuronal density and apoptosis were analyzed by immunohistochemistry using anti-NeuN and anti-active caspase 3 antibodies, respectively (Fig. 2B). Neuronal density and cell survival were not affected in the cKO cortex and hippocampus, suggesting that ventricle enlargement was not due to degeneration of the surrounding structures.

### Kidins220 cKO mice display altered dendritic and spine development in cortical and hippocampal neurons

**Fig. 3:**
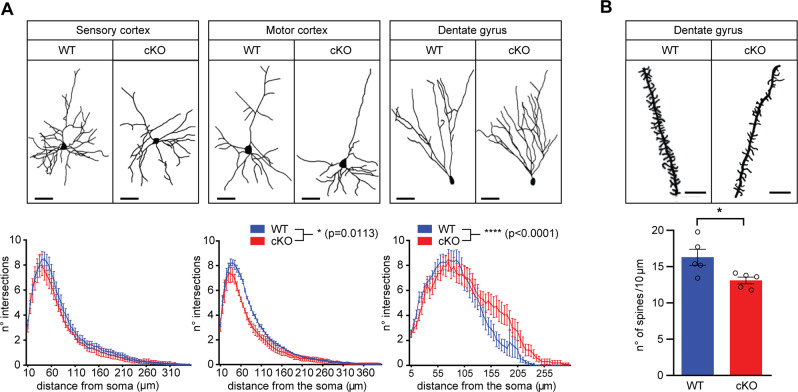
Kidins220 cKO mice display altered dendritic branching in the motor cortex and dentate gyrus, and reduced spine density in the dentate gyrus of the hippocampus. **A** Kidins220 cKO (red) and WT (blue) animals between 2M and 3M of age were perfused, brains were dissected, and processed for Golgi-Cox staining. Upper panel: representative images; scale bar, 100 µm. Lower panel: reduced dendritic arborization was observed in the motor cortex, and increased arborization in the granule cells of the hippocampus (RM-ANOVA/Holm-Šídák’s multiple comparisons test, the genotype effect is indicated. *p < 0.05; ****p < 0.0001; *n* = 3 animals per genotype from three separate litters. 3–10 cells were analysed per animal). **B** Brains were dissected from 3M-old cKO and WT animals and the dentate gyrus of the hippocampus was processed for Golgi-Cox staining. Upper panel: representative images; scale bar: 5 µm. Lower panel: quantification of the number of spines/10 μm-dendrite stretch shows a significant reduction in cKO animals compared to WT. Unpaired Student’s *t*-test, *p < 0.05; *n* = 5 animals per genotype. At least 18 dendrites from 6 different cells per animal were analysed. Circles indicate the average number of spines for each animal). All data are expressed as means ± S.E.M.

Dendritic branching was quantified by Sholl analysis in the sensory and motor cortices and in the dentate gyrus of the hippocampus of cKO and WT animals. Neurons in the cKO motor cortex had a less complex dendritic arbor, while the sensory cortex was not affected (Fig. 3A, left and middle panels). This did not impact on the general organization of the cortex, as the thickness of cortical layers was comparable between genotypes (Fig. S3). In contrast, Sholl analysis performed on the granule neurons of the dentate gyrus revealed longer and more complex dendritic ramifications in cKO neurons (Fig. 3A, right panel), accompanied by reduced spine density (Fig. 3B). The same analysis performed in +/+ and Kidins220^lox/lox^ mice showed no difference in dendrite arborization in all the areas analyzed (Fig. S4). These data show that in the adult brain, Kidins220 is involved in the maintenance of the dendritic arborization and spine development of selected neuronal populations.

### cKO mice display sex-dependent contextual memory deficits

**Fig. 4:**
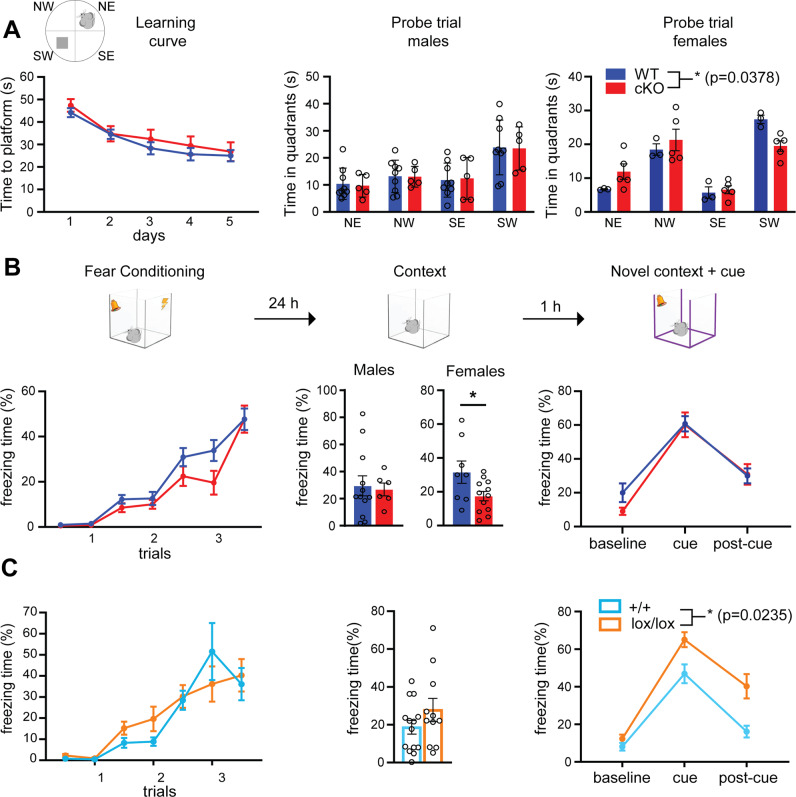
Spatial memory is impaired in cKO females and associative memory is increased in Kidins220^lox/lox^ mice. **A** Morris Water Maze in cKO mice. Left: For the analysis, the maze was divided into four quadrants: north-west (NW), north-east (NE), south-east (SE), and south-west (SW), where the platform was located. The learning curve shows the time to reach the platform during the 5 training days, which was comparable in WT and cKO mice. Middle and right: the performance during the probe trial is represented for males and females separately. The performance of male mice was comparable among genotypes, while cKO females show impaired recollection of the platform location. Values are plotted as mean ± S.E.M. and individual values are represented with circles. The trial effect (not indicated in the graph) during the training phase was tested with a Repeated Measures ANOVA (RM-ANOVA) test [*F*_4,80_ = 15.88, *p < 0.05], indicating that both genotypes are efficiently learning the task. The quadrant effect during the probe trial was tested with a two-way ANOVA test: males [*F*_3,36_ = 7.451, *p* > 0.05]; females [*F*_3,24_ = 28.95, *p < 0.05] with a significant genotype-quadrant interaction, indicated in the graph [*F*_3,24_ = 3,45, *p < 0.05, RM-ANOVA] and significant trial effect indicating different amount of time in each quadrant, not indicated in the graph for clarity. cKO (red) *n* = 10 (5 males); WT (blue) *n* = 1 (9 males). **B** Fear conditioning in cKO mice. Schematic representation of the different phases of the test (top) and corresponding quantification (bottom). The effectiveness of the conditioning is plotted as percentage time freezing. *Fear conditioning trial*. Three consecutive trials were performed (see Materials and methods section for details). The trial effect was tested with a RM-ANOVA test [*F*_6, 210_ = 57.89, ****p < 0.0001], indicating increased freezing as the trial progressed (not indicated in the graph). Both genotypes show comparable freezing behavior (genotype effect: [*F*_1,35_ = 2.81; *p* > 0.05 RM-ANOVA]). *Context trial*. This trial consisted of 5 min in the conditioning chamber in the absence of the cue and the shock. The results in this trial are represented separately for males and females. The genotype effect was tested with Student’s unpaired *t*-test; *p < 0.05. *Novel context* *+* *cue*. This trial consisted of 6 min in a new chamber during which mice were exposed to the same cue as in the fear conditioning trial. The trial effect was tested with a RM-ANOVA test [*F*_2, 74_ = 62.96, ****p < 0.0001]. No genotype effect was found during this trial: cKO mice show comparable conditioned behavior to WT mice [*F*_1,37_ = 0.112; *p* > 0.05 RM-ANOVA]. cKO *n* = 18 (6 males and 12 females), WT *n* = 20 (12 males and 8 females). **C** Fear conditioning in Kidins220^lox/lox^ mice. The experiment was performed and quantified as in **B**. *Fear conditioning trial*. The trial effect was tested with a RM-ANOVA test [*F*_6, 138_ = 19.53, ****p < 0.0001], indicating increased freezing as the trial progressed (not indicated in the graph). Both genotypes show comparable freezing behavior (genotype effect: *F*_1,23_ = 2.194; *p* > 0.05 RM-ANOVA). *Context trial*. The genotype effect was tested with Student’s unpaired *t*-test; *p* > 0.05. *Novel context* *+* *cue*. The trial effect was tested with a RM-ANOVA test (genotype effect: *F*_2,42_ = 4.1, *p < 0.05, indicated in the graph; Holm-*Š*ídák’s multiple comparisons test: **p < 0.01 in ‘cue’, ***p < 0.001 in ‘post-cue’ not indicated in the graph for clarity). Kidins220^lox/lox^ *n* = 11; +/+ *n* = 14.

To assess the impact of Kidins220 ablation on higher brain functions, a series of behavioral tests were performed. We first assessed parameters such as the coat condition, motor functions, reflexes, sensory functions, and analgesic behavior, and found no differences between WT and cKO animals (Table 1). To assess spatial memory, we performed the Morris water maze test. The performance of WT and cKO animals over the 5-day training was comparable, indicating that short-term spatial memory, motor skills, and visual recognition were preserved. However, in the probe trial, cKO females performed significantly worse than WT (Fig. 4A). To study whether Kidins220 plays a role in associative learning, we performed the fear conditioning test. During the conditioning phase, the two genotypes displayed comparable freezing, indicating that short-term associative learning was intact (Fig. 4B, left). 24 h after the conditioning, mice were re-exposed to the same context in the absence of cue (‘context’ trial), to assess contextual memory, and subsequently to a novel context in the presence of the conditioned stimulus (‘novel context + cue’ trial), to assess cue-dependent memory. Again, cKO females froze less than WT in the context trial, reinforcing the evidence of a gender-dependent impairment in contextual memory (Fig. 4B, middle). In the ‘novel context + cue’ trial, the performance of both genders in the two genotypes was comparable (Fig. 4B, right).

**Table 1:** General health assessment of cKO mice.

	WT (*n* = 8)	cKO (*n* = 8)
Appearance (%)		
Poor coat condition	0	0
Bald patches	0	0
Missing whiskers	0	0
Piloerection	0	0
Abnormal response	0	0
Motor functions		
Trunk curl (%)	100	100
Forepaw reaching (%)	100	100
Coordination and balance on beam (3 point scale)	2 ± 0	2 ± 0
Grip strength (3 point scale)	2 ± 0	2 ± 0
Reflexes (%)		
Righting reflex	100	100
Eye twitch	100	100
Ear twitch	100	100
Whisker orienting reflex	100	100
Postural reflex	100	100
Sensory functions (%)		
Visual placing	100	100
Negative geotaxis	100	100
Acoustic startle response	100	100
Analgesic behavior		
Hot plate latency (s)	145.37 ± 12.34	127 ± 15.33

We repeated selected behavioral experiments in Kidins220^lox/lox^ and +/+ animals. Kidins220^lox/lox^ animals showed increased freezing behavior in the novel context part of the fear conditioning test, while contextual memory was comparable between genotypes and genders (Fig. 4C). These results indicate that the forebrain-specific ablation of Kidins220 impacts on hippocampus-dependent functions selectively in female mice, and that the alteration of the Kidins220 splicing pattern specifically impacts on associative memory.

While performing the above-described tests, we noticed different performances of control groups. We thus directly compared WT and +/+ mice and noticed that Cre-expressing animals displayed increased freezing in the novel context phase of the fear conditioning test (Fig. S5A). These data underlie the importance of selecting the appropriate control groups.

### cKO mice display decreased anxiety-like traits

**Fig. 5:**
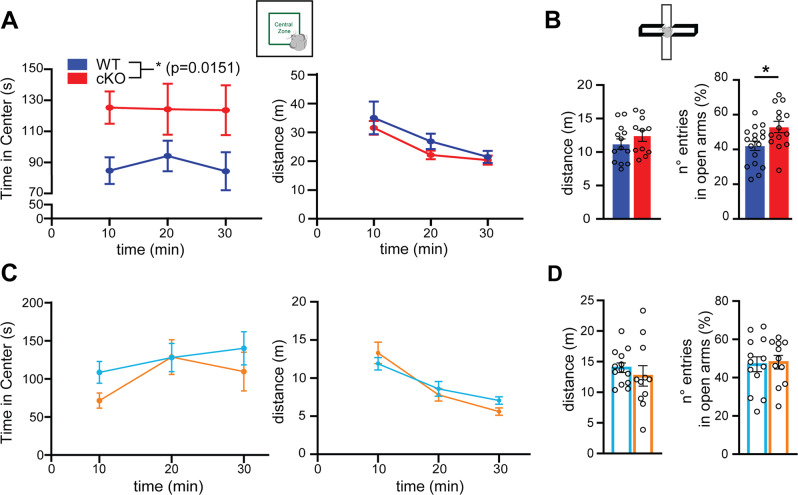
Anxiety is reduced in cKO mice. **A** Open Field in cKO mice. Schematic representation of the test (top) and quantification of the distance traveled (bottom right) and of the time spent in the central zone (bottom left). The quantification is expressed as the time (s) spent in the central zone every 10 min throughout the 30 min trial. Values are plotted as mean ± S.E.M. The genotype effect was tested with a RM-ANOVA test (*F*_1, 37_ = 4.568, *p < 0.05). cKO *n* = 19; WT *n* = 21. **B**. Elevated plus Maze in cKO mice. Schematic representation of the test (top) and quantification of the distance traveled (*bottom left*) and the *n*. of entries in the open arms during the 5 min test (bottom right). The quantification is expressed as percentage of the number of entries in the open arms over the total *n*. of entries. The genotype effect was tested with Student’s unpaired *t*-test; *p < 0.05. cKO *n* = 14; WT *n* = 17. **C** Open Field in Kidins220 ^*lox/lox*^. The experiment was performed and quantified as in **A**. The behavior is comparable among genotypes (*F*_1,21_ = 0,1164; *p* > 0.05 genotype effect, RM-ANOVA). Kidins220^lox/lox^ *n* = 9; +/+ *n* = 10. **D**. Elevated plus Maze in Kidins220 ^*lox/lox*^. The experiment was performed and quantified as in **B**. Both the distance traveled and the percentage *n*. entries in open arms are comparable among genotypes (Student’s unpaired *t*-test; *p* > 0.05). Kidins220^lox/lox^ *n* = 11; +/+ *n* = 13. In all panels, values are plotted as mean ± S.E.M. and individual values are represented with circles.

We next investigated anxiety-related phenotypes in cKO animals by the open field and elevated plus-maze tests. In the open field test, cKO animals spent more time in the central zone over the 30 min trial (Fig. 5A, left), while showing an overall motor activity similar to WT (Fig. 5A, right). In the elevated plus-maze test, cKO mice entered more times in the open arms (Fig. 5B). In these tests, no gender differences were observed. Kidins220^lox/lox^ and +/+ animals showed comparable performances in both open field (Fig. 5C) and elevated plus maze (Fig. 5D) tests with no gender differences, indicating that the anxiety-like phenotypes were attributable to the deletion of Kidins220.

We compared the anxiety-related phenotypes of the WT and +/+ groups and observed that WT mice are characterized by decreased anxiety levels and hyperactivity in the open field test (Fig. S5B), while the performance in the elevated plus-maze test was comparable (Fig. S5C).

### TrkB-dependent BDNF signaling is altered in Kidins220 cKO mice

**Fig. 6:**
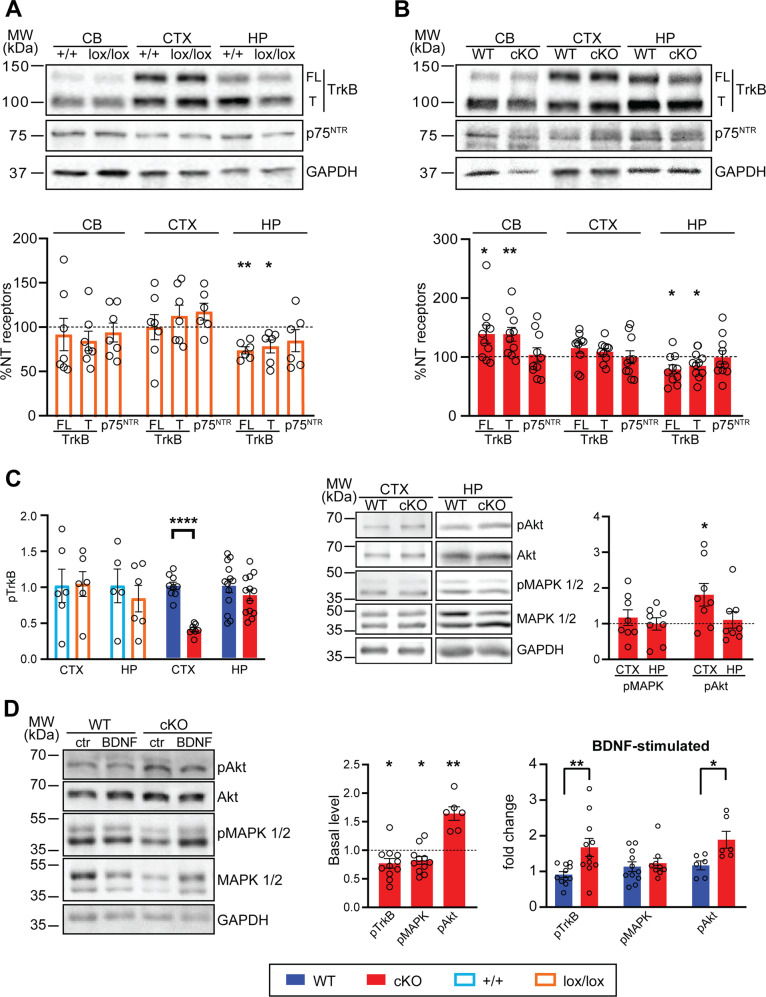
TrkB levels are reduced in the hippocampus of Kidins220^lox/lox^ and cKO mice. **A**, **B** Cerebellum (cb), cortex (ctx), and hippocampus (hp) of animals at 3 M of age were lysed and analysed by western blotting with anti-TrkB and p75^NTR^ antibodies. Top: representative immunoblots for +/+ and Kidins220^lox/lox^ (**A**) and WT and cKO (**B**) animals are shown as indicated. Bottom: quantification of immunoreactive bands for Kidins220^lox/lox^ (orange) and cKO (red), compared to the corresponding control samples, set to 100% (dashed lines). Full length and truncated TrkB levels are significantly reduced in the hippocampus of Kidins220^lox/lox^ and cKO mice and increased in the cerebellum of cKO mice. The expression of p75^NTR^ was not altered in any sample analysed. The intensity of the bands from Kidins220^lox/lox^ (orange) and cKO (red) samples were normalized to the corresponding ones from +/+ and WT samples within the same nitrocellulose membrane, respectively. One-sample Student’s *t*-test, *p < 0.05, **p < 0.01 *n* = 6–7 for +/+ and lox/lox; *n* = 10 for WT, cKO. **C** Left: phosphorylated TrkB levels were analysed by ELISA in the cortex and hippocampus of +/+ and Kidins220^lox/lox^, cKO and WT mice. P-TrkB levels were comparable in +/+ and lox/lox samples, while P-TrkB is strongly reduced in the cKO cortex. Values of lox/lox and cKO samples were normalized to values from +/+ and WT samples within the same ELISA plate. Unpaired Student’s *t*-test, ****p < 0.0001 *n* = 5–6 for +/+ and lox/lox; *n* = 9-13 for WT and cKO. Right: Phosphorylated MAPK1/2 and Akt levels were analysed by western blotting in the cortex and hippocampus of cKO and WT mice. P-MAPK1/2 levels are similar in both genotypes while P-Akt is selectively increased in the cortex of cKO animals. One sample Student’s *t*-test, *p < 0.05, *n* = 9-11 for pTrkB and pMAPK1/2, *n* = 6 for pAkt. **D** WT and cKO coronal cortico-hippocampal slices were treated with 10 ng/ml BDNF for 10 min or left untreated (Ctr). After treatment, slices were lysed and analysed for phosphorylated MAPK1/2 (Thr202/Tyr204) and Akt by western blotting, and for phosphorylated TrkB by ELISA. *Left*: for the western blot analysis, membranes were probed for phospho-MAPK1/2 and phospho-Akt, subsequently stripped and re-probed for the total amount of the same protein. *Middle*: Comparison of basal levels of phosphorylated TrkB, MAPK1/2, and Akt in untreated lysates. Values of cKO samples were normalized to the corresponding values from WT samples within the same nitrocellulose membrane or ELISA plate. One-sample Student’s *t*-test, *p < 0.05, **p < 0.01. Right: fold change of TrkB, MAPK1/2, and Akt phosphorylation upon BDNF stimulation. Values of phosphorylated proteins were first normalized to the total amount of protein, and subsequently to the untreated samples. Student’s unpaired *t*-test, *p < 0.05, **p < 0.01, *n* = 6-11 animals per genotype. In all panels, values are plotted as mean ± S.E.M, and individual values are represented with circles.

To determine whether neurotrophin signaling was altered in our mouse lines, we first quantified the amount of the BDNF receptors TrkB and p75^NTR^ in various brain areas. We found a reduction of full-length and truncated TrkB in the hippocampus of cKO and Kidins220^lox/lox^ mice and an increase of the same receptors in the cKO cerebellum, while they remain constant in the cortex of all genotypes. p75^NTR^ expression remained unchanged in all the samples analyzed (Fig. 6A,B). No differences in TrkB and p75^NTR^ expression were detected in the amygdala (Fig. S6). We measured the concentration of BDNF in brain lysates of cKO and Kidins220^lox/lox^ mice by enzyme-linked immunosorbent assay (ELISA), showing no differences in all the analyzed structures (Fig. S7). We subsequently quantified the amount of phosphorylated TrkB (pTrkB) in hippocampal and cortical lysates through ELISA. Interestingly, while no differences in pTrkB were detected in Kidins220^lox/lox^ samples compared to +/+, cKO animals showed markedly decreased pTrkB in the cortex, while the amount of pTrkB in the hippocampus was comparable to WT (Fig. 6C, left).

Given the stronger phenotype observed in cKO mice, we restricted our subsequent analysis to cKO animals and asked whether BDNF/TrkB signaling was affected by the lack of Kidins220. Basal phosphorylation levels of MAPK1/2 and Akt were assessed, showing a clear increase in basal pAkt in cKO cortical tissue (Fig. 6C, middle and right). To assess the responsiveness of the cKO brain to BDNF stimulation, coronal cortico-hippocampal slices were challenged with 10 ng/ml BDNF for 10 min and the phosphorylation levels of TrkB, MAPK1/2, and Akt were evaluated. cKO slices were characterized by reduced basal levels of pTrkB and pMAPK1/2 and increased basal levels of pAkt (Fig. 6D, middle). Upon BDNF stimulation, we observed a significant increase in pTrkB and pAkt in cKO slices, while pMAPK1/2 was comparable to WT (Fig. 6D, right).

Altogether, these experiments show that in the absence of Kidins220 the BDNF-TrkB system is affected at multiple levels: cKO mice are characterized by reduced TrkB expression in the hippocampus and reduced pTrkB levels in the cortex, as well as by altered activation of Akt under basal conditions and upon BDNF stimuli.

### BDNF signaling only partially contributes to the anxiety-like phenotype of Kidins220 cKO mice

**Fig. 7:**
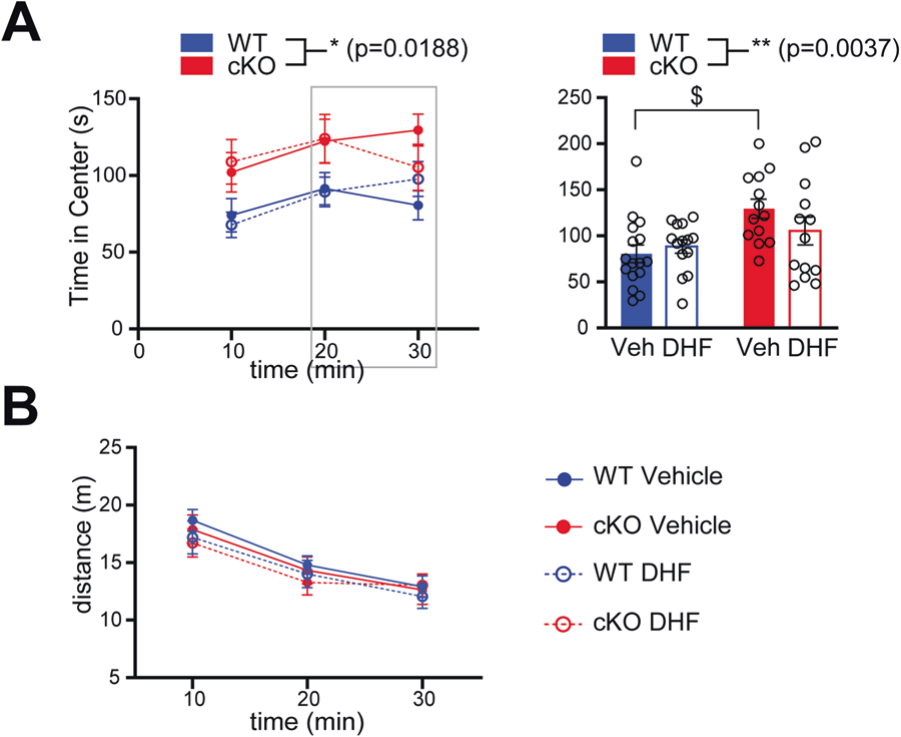
BDNF signaling only partially contributes to the anxiety-like phenotype of Kidins220 cKO mice. **A** WT and cKO mice that received DHF or vehicle in the drinking water for 4 weeks were tested in the open field test. The experiment was performed and quantified as in Fig. 5A. Left: cKO mice spent more time in the center of the arena, confirming the reduced anxiety phenotype (*F*_3,53_ = 3.623; *p < 0.05 genotype effect, RM-ANOVA). Right: the bar graph shows the time spent in the center by the four experimental groups in the last 10 min of the test. Vehicle-treated cKO mice spent significantly more time in the center compared to WT animals (*F*_1,52_ = 9.250; **p < 0.01 genotype effect, $*p* < 0.05 two-way ANOVA/Holm-Šídák’s multiple comparisons test), while no difference was detected between the DHF-treated groups. **B** The distance traveled was comparable across genotypes (*F*_3,51_ = 0.2802; *p* > 0.05 genotype effect, RM-ANOVA). WT Vehicle *n* = 16; WT DHF *n* = 15; cKO Vehicle *n* = 13; cKO DHF *n* = 13. In all panels, values are plotted as mean ± S.E.M, and individual values are represented with circles.

Given the well-established involvement of the BDNF/TrkB system in anxiety [29–36], we sought to rescue the reduced anxiety observed in the open field test by chronic administration of 7,8-DHF, a known agonist of BDNF signaling [37]. cKO mice receiving DHF showed a reduction of the time spent in the center in the last 10 min of the test (Fig. 7A), so that their performance became indistinguishable from WT mice subjected to the same treatment. However, no statistical difference between the Veh and DHF cKO groups was observed over the total time of observation. The treatment did not affect the activity of the mice, as shown by the total distance traveled by the four experimental groups (Fig. 7B). The pharmacological rescue experiments indicate that alterations in BDNF signaling system contribute only partially to the low-anxiety phenotype of cKO animals.

## Discussion

We compared Kidins220^lox/lox^ and Kidins220 cKO animals and identified changes of brain neuron morphology and reduced anxiety, partially ascribable to impairments of the BDNF/TrkB axis.

Kidins220^lox/lox^ and cKO mice display enlarged brain ventricles, similar to mouse embryos constitutively lacking Kidins220 [18, 23]. This phenotype is relevant as human fetuses bearing loss-of-function Kidins220 mutations and children affected by SINO display ventriculomegaly [3, 4]. A percentage of Kidins220^lox/lox^ mice develop hydrocephalus. Kidins220 controls AQP4 recycling via the SNX27 complex in glial cells: when Kidins220 protein levels are reduced, AQP4 enters the degradative pathway causing impaired water flux [28]. This happens in the absence of neural death (our data), confirming that the cause of hydrocephalus lies in altered cerebrospinal fluid homeostasis. Of note, we observed enlarged ventricles in Kidins220^lox/lox^ mice, in agreement with [28], and this phenotype is even more evident in cKO animals, suggesting that the removal of Kidins220 from excitatory neurons impairs the neuron-glia crosstalk.

Our behavioral tests indicate gender-dependent spatial memory deficits and reduced anxiety-like traits in cKO, but not in Kidins220^lox/lox^, mice. Spatial memory deficits were observed also in heterozygous female mice [38]. The mechanisms underlying such sexual dimorphism are not known but they likely include the interaction between estrogens and BDNF. Indeed, BDNF deficiency impacts differently on male and female mouse behavior [[39] and reviewed in [40]], and estradiol levels modulate spatial memory through BDNF in female mice [41]. The brain circuits underlying anxiety include the amygdala, medial prefrontal cortex and hippocampus [42]. Kidins220 acts as a ‘brake’ for neural activity, as in its absence increased basal synaptic transmission [43] and enhanced LTP [17] are observed; moreover, Kidins220 contributes to BDNF-and aging-dependent plasticity [18, 44]. One underlying mechanism is the Kidins220-mediated targeting of GluA1 to the post-synaptic membrane so that in its absence, more GluA1-lacking AMPARs are present, leading to increased excitability [43]. We hypothesize that the increased excitatory drive of cortico-hippocampal circuits in mutant animals leads to the observed reduction of anxiety; indeed, widespread activation of CaMKII-positive neurons leads to a reduction of anxiety levels [45]. We also observed a specific enhancement of associative memory in Kidins220^lox/lox^ animals. The role of Kidins220 isoforms in plasticity and memory is still not understood, but alterations of neurotrophic signaling may play a role [27].

We observed reduced levels of full-length and truncated TrkB in the hippocampus of cKO and Kidins220^lox/lox^ mice, accompanied by reduced spine density and altered morphology of dentate gyrus granule cells in cKO. Of note, the latter defect was not observed in Kidins220^lox/lox^ mice, indicating that it is selectively induced by the absence of Kidins220 in excitatory neurons. Granule cells strongly depend on the BDNF/TrkB system [[46–48] and reviewed in [49]]; thus, our data suggest that within the hippocampus Kidins220 modulates TrkB levels to control dendritic arborization and spine stability. Since the hippocampus is also involved in anxiety behaviors [42, 46], the observed abnormalities could contribute to the altered anxiety levels of cKO animals. Reduced TrkB levels in the absence of Kidins220 are observed also in vitro [22], however, the cellular mechanisms are not known. In the absence of Kidins220 TrkB clustering is reduced [24]. Indeed, Kidins220 participates to the sorting of early endosomes to the degradative versus recycling fate [50] and in the absence of Kidins220, endocytosed Trk receptors may be more rapidly or preferentially directed toward the degradative pathway, similar to AQP4 [28]. Unexpectedly, we observed increased TrkB levels in the cKO cerebellum. Cerebellar development relies on BDNF-TrkB [48, 51–54]; in our mice, the cerebellum is not targeted by the CaMKII promoter, so compensatory mechanisms may be at play, in response to the reduced activation of TrkB in the cortex (see below). The underlying mechanisms are of relevance – and will be the subject of our future studies – since SINO manifests itself chiefly with motor symptoms. Another symptom of SINO is obesity, and indeed the CaMKII-Cre-driven deletion of BDNF induces obesity [29]. Our animals develop normally in size and body weight and in this case, a contribution of glial Kidins220 is envisaged, as Kidins220 plays a role in astroglia metabolism [22].

Animals expressing reduced Kidins220 levels display reduced dendritic arborization [24, 25]. We do not see such defects in the somatosensory cortex and cKO mice do not show any impairment in sensory abilities and reflexes. We observed a small genotype effect in motor cortex, which did not impact on the motor ability of the animals, however, we cannot exclude subtle motor deficits. The discrepancy between our findings and previous data is likely due to the different models. The animals analyzed by Wu and colleagues [24, 25] display reduced Kidins220 expression in all brain cells, including neurons, glial and microglial cells, while in utero electroporation downregulates Kidins220 expression since embryonic development [24]. Our cKO animals lack Kidins220 only in forebrain excitatory neurons from the second postnatal week. In light of our data, therefore, the dendritic arborization defects observed in the above-described models should be re-interpreted as due to a combination of factors, including non-cell autonomous effects deriving from altered glial cell physiology – as Kidins220 plays an important role in astroglia [21, 22] – as well as alterations of developmental processes.

cKO mice show a striking reduction of phosphorylated TrkB in the cortex, where TrkB protein levels are normal. Remarkably, pTrkB levels were preserved in Kidins220^lox/lox^ animals, indicating that this phenotype is specifically caused by Kidins220 deletion. However, cKO tissue is more reactive upon BDNF stimulation. As discussed above, this could be due to altered TrkB localization and/or recycling. We previously [18] showed that membrane TrkB levels are not altered in Kidins220 KO neurons in culture. Rather, in the absence of Kidins220, the receptor may redistribute between membrane sub-compartments, such as lipid rafts, in a way that enhances its availability for BDNF sensing and subsequent activation. This is a possibility since Kidins220 is also associated with the raft compartment [55]. Moreover, Akt is over-activated both under basal conditions and upon BDNF stimulation. The PI3K-Akt cascade controls dendritic arborization through mammalian target of rapamycin (mTOR)-dependent protein synthesis [56] and within the hippocampus, BDNF-dependent Akt activation is required for spine morphogenesis and plasticity [57]. Of note, Kidins220 controls cortical neuron morphogenesis mainly through PI3K-Akt [24]. The overactivation of the Akt pathway in the presence of chronically reduced TrkB phosphorylation could be the expression of compensatory mechanisms.

Does the low-anxiety phenotype observed in cKO mice depend on alterations of the BDNF-TrkB system? The role of BDNF in anxiety is debated [58]. Mutant mouse lines with reduced levels of BDNF show increased [29, 30] or unaltered [31] anxiety, and increasing BDNF levels also increases anxiety [32, 33]. The lack of Kidins220 has different impacts on BDNF/TrkB signaling in different brain areas and chronic activation of TrkB recovers the phenotype only partially, indicating that alterations of BDNF pathways do play a role, but other molecular players are involved.

As a last remark, our study underlies the importance of evaluating the impact of all transgenic modifications, even those assumed to be ‘neutral’ such as the insertion of lox sites and Cre expression, on mouse physiology and behavior.

In this work we laid the basis for understanding SINO syndrome, identifying alterations in the neurotrophic system as one factor contributing to the pathology. Indeed, one pathogenic *KIDINS220* variant shows reduced binding to TrkA [9], suggesting neurotrophin pathways as potential targets for future therapeutic approaches.

## Materials and methods

### Generation of Kidins220 CaMKII-Cre driven conditional knockout animals

To achieve the forebrain-specific deletion of Kidins220, animals bearing the floxed Kidins220 gene [Kidins220(lox) strain, [18, 23]] were crossed with animals expressing the Cre recombinase gene under the control of the Calcium/calmodulin-dependent kinase II alpha promoter [CaMKIIα strain, stock #B6.Cg-Tg(Camk2a-cre)T29-1Stl/J, Jackson Laboratories, Bar Harbor, ME]. Both the Kidins220(lox) and the CaMKIIα colonies were on the C57BL/6 background and were maintained separately. For the experiment with the td-tomato animals [td-tomato(lox) strain, stock #B6.Cg-Gt(ROSA)26Sortm14(CAG-tdTomato)Hze/J, Jackson Laboratories], experimental (td-tomato^lox/+;Cre/+^) and control (td-tomato^+/+;Cre/+^) animals were obtained from [td-tomato^lox/+^ X CaMKII-Cre^Cre/Cre^] breeding couples. For experiments with the Kidins220(lox) colony, Kidins220^lox/lox^ and control (Kidins220^+/+^, +/+) animals were derived from [Kidins220^+/lox^ X Kidins220^+/lox^] breeding couples. Conditional knockout (Kidins220^lox/lox;Cre/+^, cKO), heterozygous (Kidins220^lox/+;Cre/+^, Het) and control (Kidins220^+/+;Cre/+^, WT) mice were derived from [Kidins220^+/lox;+/+^ X Kidins220^+/lox;Cre/Cre^] breeding couples. Mice were maintained on a 12:12 h light/dark cycle (lights on at 7 a.m.). The temperature was maintained at 21 ± 1 °C, relative humidity (60 ± 10%). Animals were provided drinking water and a complete pellet diet (Mucedola, Settimo Milanese, Italy) ad libitum. Mouse genotypes were determined at weaning (at P21) by PCR or RT-PCR on tail samples. Genotyping of the Kidins220(lox) and cKO alleles was conducted as reported previously [18, 23], while genotyping of the Cre transgene was conducted following the procedure indicated by Jackson Laboratories. Mice were weaned into cages of same sex pairs. All experiments were carried out in accordance with the guidelines established by the European Communities Council (Directive 2010/63/EU of 22 September 2010) and were approved by the Italian Ministry of Health.

### Western blotting

Brain tissue and slices were homogenized in RIPA buffer (50 mM Tris-HCl pH 7.4, 150 mM NaCl, 2 mM EDTA, 1% NP40, 0.1% SDS) containing protease and phosphatase inhibitors (complete EDTA-free protease inhibitors, Roche Diagnostic S.p.A., Monza, Italy; serine/threonine phosphatase inhibitor and tyrosine phosphatase inhibitor, Sigma Aldrich, Milano, Italy). After centrifugation at 16,000 × *g* for 15 min at 4 °C, protein concentration in the supernatants was quantified by using the BCA Protein Assay kit (Thermo Fisher Scientific, Waltham, MA). Protein lysates (20 μg /sample) were separated by SDS-PAGE by using 6–12% polyacrylamide gels and transferred on nitrocellulose membranes. The following primary antibodies were used: the polyclonal anti-Kidins220 antibody was a kind gift of Dr. Iglesias Vacas [Instituto de Investigaciones Biomédicas “Alberto Sols” (CSIC-UAM) and CIBERNED (ISCIII), Madrid, Spain], rabbit monoclonal anti-GAPDH (14C10, #2118, Cell Signaling Technology, Leiden, The Netherlands), rabbit polyclonal anti-TrkB (#07-225, Merck-Millipore, Darmstadt, Germany), rabbit polyclonal anti-p75^NTR^ (#G323A, Promega, Milano, Italy), rabbit monoclonal anti-phosphorylated MAPK1/2 (Thr202/Tyr204, #4377, Cell Signaling Technology), rabbit polyclonal anti-MAPK1/2 (#06-182, Merck-Millipore), rabbit monoclonal anti-phosphorylated Akt [Ser473 (193H12) #4058, Cell Signaling Technology], rabbit polyclonal anti-Akt (#9272, Cell Signaling Technology), and rabbit polyclonal anti-actin (#A2066, Sigma Aldrich). After incubation with primary antibodies, membranes were incubated with HRP-conjugated secondary antibodies (goat anti rabbit #31460, Thermo Fisher Scientific) and revealed by using the ChemiDoc MP Imaging System (Biorad, Hercules, CA). Immunoreactive bands were quantified by using the ImageJ software. Intensity of phosphorylated proteins was normalized to the total amount of the corresponding protein.

### Analysis of brain anatomy

Kidins220 cKO and WT animals between 2 and 3M of age were perfused transcardially with 4% PFA, their brains dissected, post-fixed overnight in the same fixative solution and cryoprotected in 20% then 30% sucrose solutions. In all, 80-µm-thick sections were prepared using a freezing microtome (Microm HM 450, Thermo Fisher Scientific), stained with the Hoechst nuclear stain (#B2261, Sigma), and mounted on slides with Mowiol. Sections were imaged using a Leica SP5 confocal microscope with a ×10/0.45 objective. Cortical thickness, size of hippocampus, area of lateral ventricles, and area of third ventricle were measured using the ImageJ software.

### Immunohistochemistry

Mouse brains were dissected and fixed overnight in 4% PFA. They were then cryoprotected in 20% then 30% sucrose, embedded in OCT, frozen in isopentane (−55 °C), and stored at −80 °C. Sagittal sections (10 μm) were cut with a cryostat and stored at −20 °C before immunostaining. Sections were rehydrated in phosphate-buffered saline (PBS) for 5 min and then fixed and permeabilized with 70% methanol/30% acetone for 15 min at −20 °C. After several washes in PBS, slices were blocked for 30 min in PBS containing 1% BSA and incubated with primary antibodies diluted in PBS-1% BSA for 2 h at room temperature (RT). The following primary antibodies were used: rabbit polyclonal anti-active caspase 3 (#AF835, R&D Systems, Minneapolis, MN), rabbit polyclonal anti-NeuN (#MAB377, Merck-Millipore), rabbit polyclonal anti Cux1 (#M222, Santa Cruz Biotechnology, Dallas, TX, US), rat monoclonal anti-CTIP2 (#AB18465, Abcam), and rabbit polyclonal anti-FoxP2 (#D55H9, Cell Signaling Technology). After washes in PBS containing 0.25% Tween-20, slices were incubated with secondary antibodies diluted in PBS-1% BSA for 45 min at RT, thoroughly washed and mounted on glass slides with Mowiol. Fluorescently conjugated secondary antibodies were from Molecular Probes (Thermo-Fisher Scientific; Alexa Fluor 488, #A11029; Alexa Fluor 647, #A21450). Hoechst (#B2261, Sigma-Aldrich) was used to stain nuclei. Sections were imaged using a Leica SP8 confocal microscope with a 40x/1.3 oil immersion objective. Quantification of the number of NeuN-positive and active caspase 3-positive cells in the hippocampus and the somatosensory cortex was performed using the ImageJ software.

### Golgi-Cox staining for Sholl analysis

The Golgi-Cox solution was prepared through the following protocol: 5 volumes of solution A [5 g/100 ml potassium dichromate (K_2_Cr_2_O_7_) in deionized H_2_O (DW)] were mixed with 5 volumes of solution B [5 g/100 ml mercuric chloride (HgCl_2_) in DW] and this mix was stirred for 30 min. Four volumes of solution C [5 g/100 ml potassium chromate (K_2_CrO_4_) in DW] were mixed with 10 volumes of DW and gently stirred. Then, solution C was added to the mix A + B slowly and the Golgi-Cox solution (A + B + C) was stirred for 30 min and incubated during 5 days in a glass bottle in the dark. Before use, the solution was filtered. 3M-old animals were perfused transcardially with saline [0.9% NaCl in DW]. Brains were immediately dissected and incubated in Golgi-Cox solution for 39 h at 37 °C. They were then incubated in a 20% sucrose solution for at least 2 days at 4 °C. In total, 150–200µm-thick slices were prepared with a vibratome (5100 mz Campden Instruments LTD, England) in a 6% sucrose solution protected from light and mounted on 2% gelatin-coated glass slides. After drying, the Golgi-Cox staining was developed using the following protocol: 33% ammonium hydroxide 30 min, sodium thiosulfate 1% in DW 30 min, 50% EtOH 1 min, 70% EtOH 1 min, 95% EtOH 1 min, 100% EtOH 5 min [2x], Solution X (1/3 chloroform, 1/3 xylene, 1/3 EtOH) 15 min, Xylene 15 min, then mounting on coverslip with Mowiol.

### Golgi Cox Staining for spine density assessment

For this analysis a commercial kit (FD Rapid GolgiStaining™ Kit FD Neurotechnologies Cat. No. PK401A) was used and the fixing solution was prepared 24 h before collecting the brains. The brains were taken without perfusion, washed with milliQ water and incubated in 5 ml of the fixing solution. The following day the solution was replaced with fresh one. Samples were protected from light and incubated in the dark for 10 days then in washing solution for 3–4 days before cutting. Brains sections were prepared using a vibratome (Campden Instruments 5100 mz); the vibratome’s bath was filled up with a solution of 5% potassium chromate (K_2_CrO_4_). Sections (120 μm) were collected on 2% gelatin-coated slides and were left to dry in the dark O/N at RT. Subsequently, slides were washed twice with milliQ water for 4 min and incubated for 10 min in a solution made by 1 part of solution D, 1 part of solution E and 2 parts of milliQ water. Slides were then washed twice with milliQ water for 4 min and then dehydrated by increasing the concentration of ethanol (50–75–95% ethanol/H_2_O), for 4 min each. Last, four washes with 100% ethanol were performed. Sections were then incubated three times in Xylene for 4 min and mounted with Mowiol. Confocal stacks were acquired using a confocal microscope Leica TCS SP8 with a ×63 oil-immersion objective and with 1 μm distance between focal planes. At least three slices per animal were taken into consideration. In each slice at least three dentate gyrus neurons were chosen and for each one at least three dendrites were selected. Spines were counted over a 30–40 µm dendrite stretch starting 20 µm from the cell body. Results are expressed as the average number of spines in a 10-µm-dendrite stretch.

### Slice stimulation

Coronal cortico-hippocampal slices (300 µm thickness) were cut from the brains of 3M-old animals using a vibratome. Slices were cut at 4 °C in a solution containing: 10 mM NaCl, 25 mM NaHCO_3_, 2.5 mM KCl, 10 mM glucose, 1.25 mM NaH_2_PO_4_, 195 mM sucrose, 2 mM sodium pyruvate, 1 mM CaCl_2_, and 2 mM MgCl_2_, then left to recover for 60 min at RT in normal artificial cerebrospinal fluid (ACSF, 125 mM NaCl, 25 mM NaHCO_3_, 25 mM glucose, 2.5 mM KCl, 1.25 mM NaH_2_PO_4_, 2 mM CaCl_2_, and 1 mM MgCl_2_). After recovery, slices were incubated in normal ACSF or ACSF containing 10 ng/ml BDNF for 10 min. Slices were constantly bubbled with carbogen (95% O_2_/5% CO_2_) throughout the experiment. After incubation, lysates were prepared from slices as described above and then subjected to western blot or ELISA analysis.

### Enzyme-linked immunosorbent assay

BDNF protein concentration in brain lysates was measured with the BDNF Emax® ImmunoAssay System (Promega) according to manufacturer’s instructions. The levels of phosphorylated TrkB were measured by sandwich ELISA assay using the monoclonal anti-phosphotyrosine (clone 4G10, #05-321, Merck-Millipore) and the polyclonal anti-TrkB (#07-225, Merck-Millipore) as capture and detection antibodies, respectively. Anti-rabbit immunoglobulin G coupled to horseradish peroxidase (#31460, Thermo Fisher Scientific) was used as secondary antibody. After incubation with TMB One solution (Promega) absorbance at 450 nm was measured on a Tecan Infinite F500 plate reader.

### Behavioral tests

All behavioral tests were performed on Kidins220 cKO or Kidins220^lox/lox^ animals and their WT and +/+ littermates, between 3 and 5 M of age. The animals were maintained in regular cages with at least 2 mice per cage and food and water ad libitum. All the animals were subjected to handling by the experimenter, 5 min per day, every day for 1 week prior to the tests and a 1-h habituation in the experimental room was performed before all tests. Males and females were tested separately, cleaning the apparatus with 70% ethanol between different animals, as well as between different trials. Whenever possible, mice were subjected to more than one behavioral procedure; in this case, they were subjected to the tests in the following order: open field, elevated plus maze, and fear conditioning. The Morris water maze test was performed on a separate group of animals.

#### Morris Water Maze test

Mice were placed for 1 h under red light in the experimental room, in order to optimize their visual skills. A tank (120 cm in diameter) was filled up to 2/3 of its volume with translucent water prepared by mixing water with biological white paint. The water was maintained at 22-24 °C through the tests. Spatial cues were placed on the walls of the room for helping mice orientation. Each training trial lasted a maximum of 60 s. Each animal was subjected to a 4-trial training per day, for 5 consecutive days. During this time, a transparent plastic platform was always maintained in the same quadrant (south-west). If the mouse failed to find the platform after 60 s, the experimenter would situate the animal on the platform. The probe test was performed the day after the last training trial; in this case, the platform was removed and the mice were left free to explore the different quadrants for 60 s. The test was recorded by an overhead camera and the Stoelting ANY-maze software was used to store the video and calculate general parameters like swimming speed and distance covered during each trial. The time taken to reach the platform in each trial and the latency during the probe test were scored manually.

#### Fear conditioning test

(i) Conditioning phase. A closed 40 cm × 40 cm chamber with transparent walls and an electrical grid as a floor was used for the conditioning. Each animal was put into the chamber and left to habituate to the novel environment for 2 min, after which the conditioning protocol was performed, which consisted of three consecutive trials, for a total of 510 s. A single trial consisted of an auditory cue (i.e., a 75 dB sound played at a frequency of 4 kHz) played for 30 s; the electrical foot shock (0.7 mA) was administered during the last 2 s of the tone presentation, and terminated together with the tone. A single trial was followed by an inter-trial interval (ITI) of 90 s. We used the TSE Multiconditioning System, *FCS v9.02* or *Shuttle 4.07*. (ii) Context phase. 24 h after the conditioning phase, the animals were tested in the conditioning chamber for 5 min without the tone or the foot shock. The environmental conditions were kept identical to the training session. (iii) Novel context + cue phase. 1 h after context testing, the animals were tested for 6 min in a novel environment, i.e., a 40 cm×40 cm chamber with black opaque walls, with the grid floor covered with a gray plastic cover and a filter paper embedded in 20 µl of apricot essential oil. After 2 min habituation to the novel context, the animals were exposed for 2 min to the same cue played during the conditioning phase, after which they were monitored for 2 additional min. The ANY-maze software was used for recording and storing the videos. The freezing behavior was scored manually by the experimenter.

#### Open Field test

Basic locomotor activity was tested by placing each animal in a large square open arena (45 cm × 45 cm x 45 cm) surrounded by non-transparent sides for 30 min under red light. The mice were situated in the center of the maze at the beginning of the test, and their movements recorded by an overhead camera. Videos were stored, tracked and analyzed by the Stoelting ANY-maze software. The total distance covered was calculated, as well as the time spent by each animal in a square 20 cm × 20 cm zone determined in the center of the arena (marked as the central zone).

#### Elevated Plus maze test

The elevated plus maze is a plus-shaped maze with four equal-sized arms (30 cm×15 cm), two of which lack sides, while the other two are closed by 15 cm high walls. The maze is elevated by 70 cm from the ground level. The test was performed under red light and during the experiment the experimenter was present in the room. Mice were placed in the middle of the cross, and their movements monitored for 5 min. The test was recorded by an overhead camera and the Stoelting ANY-maze software was used to store the video and calculate the number of entrances and the time spent in each arm.

### Statistical analysis

Data are presented as means ± S.E.M. throughout the text. The distribution of the data was assessed using the D’Agostino–Pearson normality test. When comparing two groups unpaired two-sided Student’s *t* test and one-sample t-test were used, and equality of variances tested through the F test. When more than two groups were compared, one-way ANOVA or repeated measures ANOVA followed by the Bonferroni’s post-hoc multiple comparison test were performed to assess significance as indicated in figure legends, and equality of variances tested through the Brown–Forsythe’s and Bartlett’s test. Alpha levels for all tests were 0.05% (95% confidence intervals). No predictive statistical methods were used to predetermine sample sizes; however, we adopted sample sizes (indicated in figure legends) in the same range of those previously reported in the literature for similar experiments. No randomization method was followed to allocate samples/animals to the various experimental groups. Investigators were not blinded to group allocation, but were blinded when assessing the outcome of the experiments. The ROUT method with *Q* = 1% was used to identify outliers for exclusion from analysis. All statistical procedures were performed using GraphPad Prism 6 software (GraphPad Software, Inc).

## Supplementary information

Supplementary Figure Legends

Supplementary Figure 1

Supplementary Figure 2

Supplementary Figure 3

Supplementary Figure 4

Supplementary Figure 5

Supplementary Figure 6

Supplementary Figure 7

## Data Availability

The datasets used and/or analyzed during the current study are available from the corresponding author on reasonable request.
