# Supplementary material for: Kidins220/ARMS modulates brain morphology and anxiety-like traits in adult mice
Source: Cell Death Discov. 2022 Feb 9;8:58. doi: 10.1038/s41420-022-00854-4 (PMC8828717; doi:10.1038/s41420-022-00854-4)
Supplement: Supplementary file 1 — Supplementary Figure Legends [file 41420_2022_854_MOESM1_ESM.docx]

Kidins220/ARMS Modulates Brain Morphology and Anxiety-Like Traits in Adult Mice

Amanda Almacellas-Barbanoj^1¶*^, Martina Albini^1,2*^, Annyesha Satapathy^1*^, Fanny Jaudon^1#^, Caterina Michetti^1,3^, Alicja Krawczun-Rygmaczewska^1,5^, Huiping Huang^4^, Francesca Manago’^4^, Francesco Papaleo^4^, Fabio Benfenati^1,3^ and Fabrizia Cesca^1,5§^

^1^Center for Synaptic Neuroscience and Technology, Fondazione Istituto Italiano di Tecnologia, 16132 Genova, Italy

^2^Department of Experimental Medicine, University of Genova, 16132 Genova, Italy

^3^IRCCS Ospedale Policlinico San Martino, 16132 Genova, Italy

^4^Genetics of Cognition Laboratory, Neuroscience area, Istituto Italiano di Tecnologia, via Morego, 30, 16163 Genova, Italy.

^5^Department of Life Sciences, University of Trieste, 34127 Trieste, Italy

^*^These authors contributed equally to this work

^¶^Present address: Clinical & Experimental Epilepsy, UCL Queen Square Institute of Neurology, London, UK

^#^Present address: Department of Life Sciences, University of Trieste, 34127 Trieste, Italy

^§^Corresponding author:

Fabrizia Cesca:

Department of Life Sciences, University of Trieste

Building Q, room 216 - via L. Giorgieri, 5 - 34127 Trieste (Italy) e-mail: fcesca@units.it; Ph. +39 0405588727

**SUPPLEMENTARY MATERIAL**

**Figure S1. Impact of Cre expression on Kidins220 protein levels.** Animals were sacrificed at 3M. Brains were dissected into cerebellum (cb), cortex (ctx) and hippocampus (hp), lysed and analysed by western blotting with anti-Kidins220 antibodies and anti-calnexin to verify equal loading. **A.** *Left:* representative immunoblots for +/+ and WT animals. *Right*: quantification of immuno reactive bands for WT, compared to the +/+ samples within the same nitrocellulose membrane, set to 100%. No difference in Kidins220 expression were detected between the two genotypes. Unpaired Student’s *t*-test; p>0.05, n = 6-7 for +/+ and WT. **B.** *Left*: Quantification of immunoreactive bands for lox/lox (orange) and cKO (red), compared to the corresponding control samples (dashed lines). *Right*: the intensity of the bands from lox/lox and cKO samples were normalized to the intensity of their respective controls within the same nitrocellulose membrane. Statistical differences were tested by one sample Student’s *t*-test; significance levels are reported in the table. Normalized values are plotted as means ± S.E.M. and individual values are represented with circles. **p<0.01, ***p<0.001; ****p<0.0001; n: Cerebellum: lox/lox 9, cKO 10; Cortex: lox/lox 8, cKO 9; Hippocampus: lox/lox 9, cKO 9.

**Figure S2. Time- and brain area-specific expression pattern of the CaMKII promoter.** CaMKII-Cre mice were crossed with td-tomato^lox/lox^ animals and the pups were perfused at different postnatal stages up to 1M, as indicated. Wild type age-matched animals were used as controls for background fluorescence (not shown). Scale bars, 500 μm.

**Figure S3. Cortical layer thickness is not altered in Kidins220 cKO animals.** The various cortical layers were identified on coronal brain slices from cKO and WT animals between 2M and 3M of age by immunohistochemical staining with layer-specific markers, i.e., Cux1 for layers II-IV, CTIP2 for layer V, and Foxp2 for layer VI. **A.** Representative images of the different stainings in the motor (top) and sensory (bottom) cortex. Scale bar, 500 μm. **B.** Layer width was quantified using ImageJ. No significant differences were found (Student’s *t*-test, p>0.05, n = 3 animals per genotype). Values are plotted as mean ± S.E.M, and individual values are represented with circles.

**Figure S4. Kidins220 lox/lox mice do not display altered dendritic branching in the cortex and dentate gyrus.** Brains of Kidins220 lox/lox (orange) and +/+ (light blue) animals at 3M of age were dissected and processed for Golgi-Cox staining. No differences in dendritic arborization were observed in the motor and sensory cortices, and in the granule cells of the hippocampal dentate gyrus. All data are expressed as means ± S.E.M. Statistical analysis was performed using the RM-ANOVA/Holm-Šídák’s multiple comparisons tests. p>0.05 (n=3 animals per genotype from 3 separate litters. 2-16 cells were analysed per animal).

**Figure S5. Impact of Cre expression on mouse behaviour. A**. *Fear conditioning*. Results from +/+ (n = 14, cyan) and WT (n = 21, blue) are shown. The amount of time the mice spent freezing during each phase of the experiment is shown as % freezing time. In the novel context + cue phase, WT froze more than +/+ (genotype effect: F_1,_ _31_ = 5.619 *p <0.05, RM-ANOVA). **B**. *Open field.* Results from +/+ (n = 12, cyan) and WT (n = 21, blue) are shown. The amount of time the mice spent in the central zone and the distance travelled during the test are plotted, divided into 10 min bins. +/+ mice spent consistently more time in the central zone (genotype effect: F (1, 29) = 6.098 *p < 0.05, RM-ANOVA), but covered less distance than the WT (genotype effect: F (1, 29) = 17.06 ***p < 0.001, RM-ANOVA). **C**. *Elevated plus maze.* Results from +/+ (n = 13 cyan) and WT (n = 17 blue) are shown. The % entries in the open arms and the % time spent in open arms are comparable between the two genotypes (p>0.05, Student’s *t*-test). In all panels, values are plotted as mean ± S.E.M. and individual values are represented with circles.

**Figure S6. TrkB and p75^NTR^ receptor levels in the amygdala are not affected in the absence of Kidins220.** Amygdalas of animals at 3M of age were lysed and analyzed by western blotting with anti-TrkB and p75^NTR^ antibodies. β-actin immunoreactivity was used to verify equal loading. *Left*: representative immunoblots for +/+ and lox/lox, WT and cKO animals, as indicated. *Right*: quantification of immunoreactive bands for the various genotypes. Unpaired Student’s *t*-test, p>0.05 (n = 5-7 for each genotype).

**Figure S7. BDNF levels are comparable in the brain of +/+, Kidins220^lox/lox^, WT and cKO mice.** An ELISA assay was performed on lysates from cortex, hippocampus, cerebellum and striatum in Kidins220^lox/lox^ compared to +/+ (**A**) and in cKO compared to WT (**B**). Pg/ml of protein are plotted as mean ± S.E.M and individual values are represented with circles. Unpaired Student’s t-test; p>0.05, n=6-7. +/+ (light blue), Kidins220^lox/lox^ (orange), WT (blue) and cKO (red).
