# Supplementary figures and images for: Kidins220/ARMS modulates brain morphology and anxiety-like traits in adult mice
Source: Cell Death Discov. 2022 Feb 9;8:58. doi: 10.1038/s41420-022-00854-4 (PMC8828717; doi:10.1038/s41420-022-00854-4)

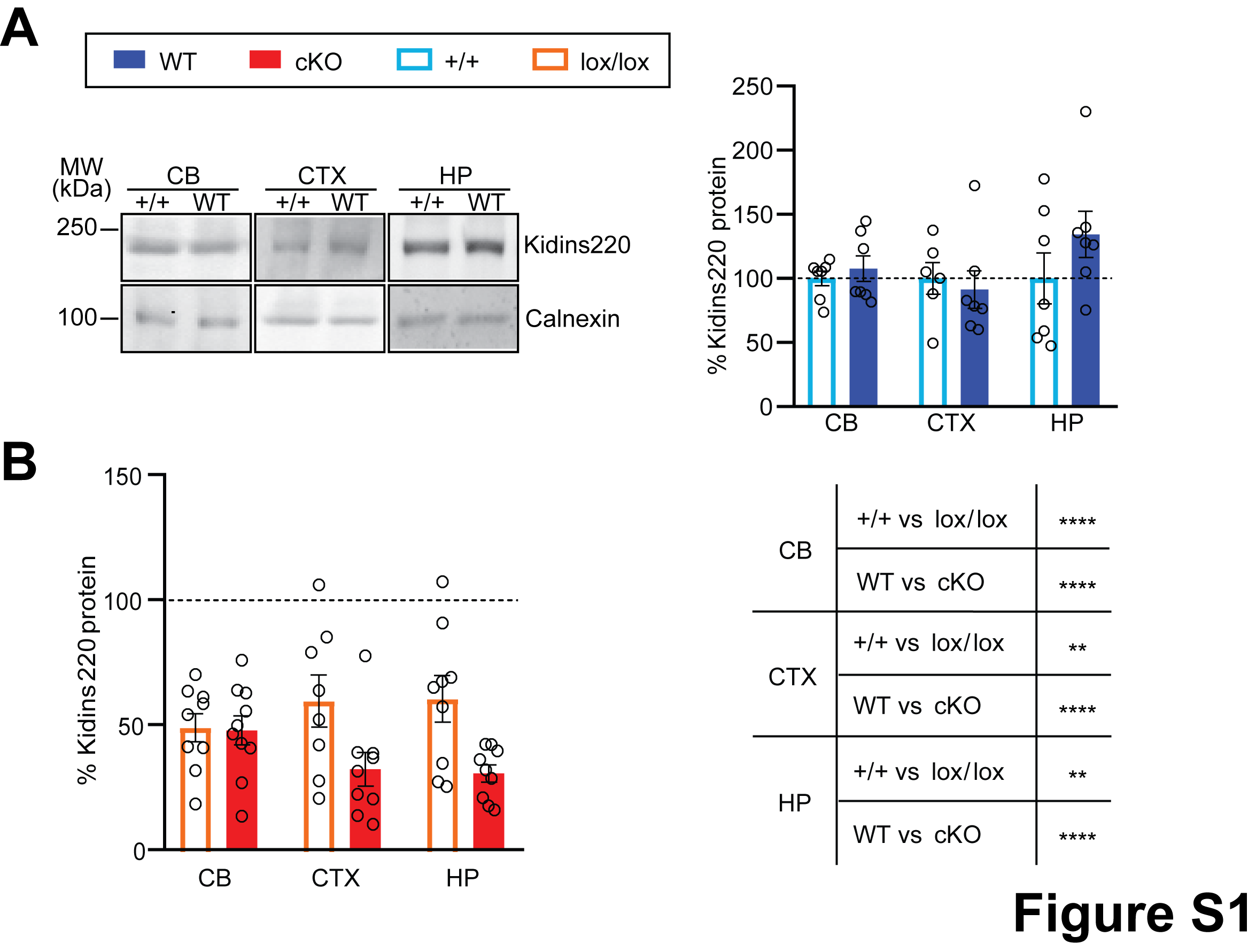

Supplement: Supplementary file 2 — Supplementary Figure 1 [file 41420_2022_854_MOESM2_ESM.tif]

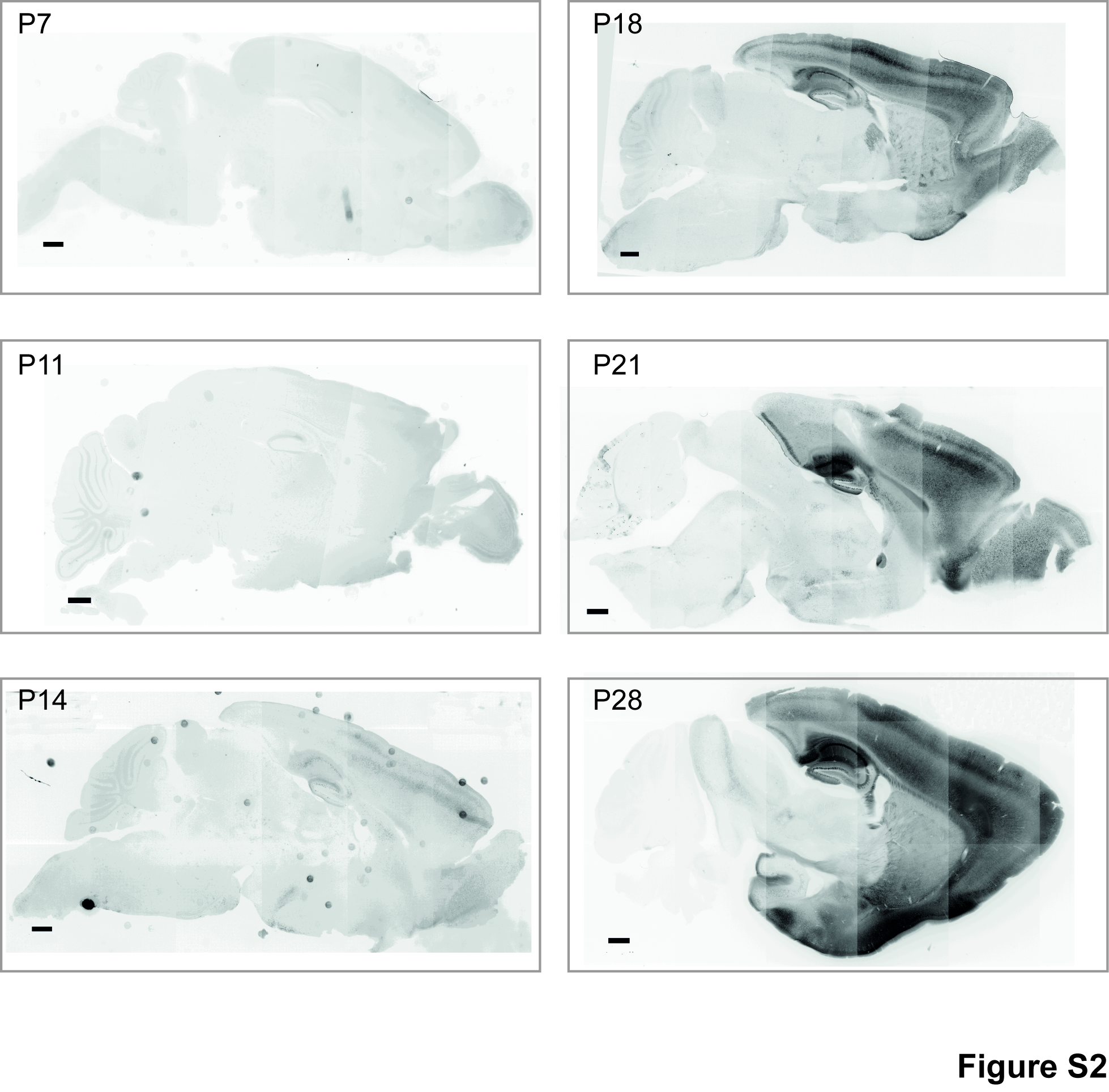

Supplement: Supplementary file 3 — Supplementary Figure 2 [file 41420_2022_854_MOESM3_ESM.tif]

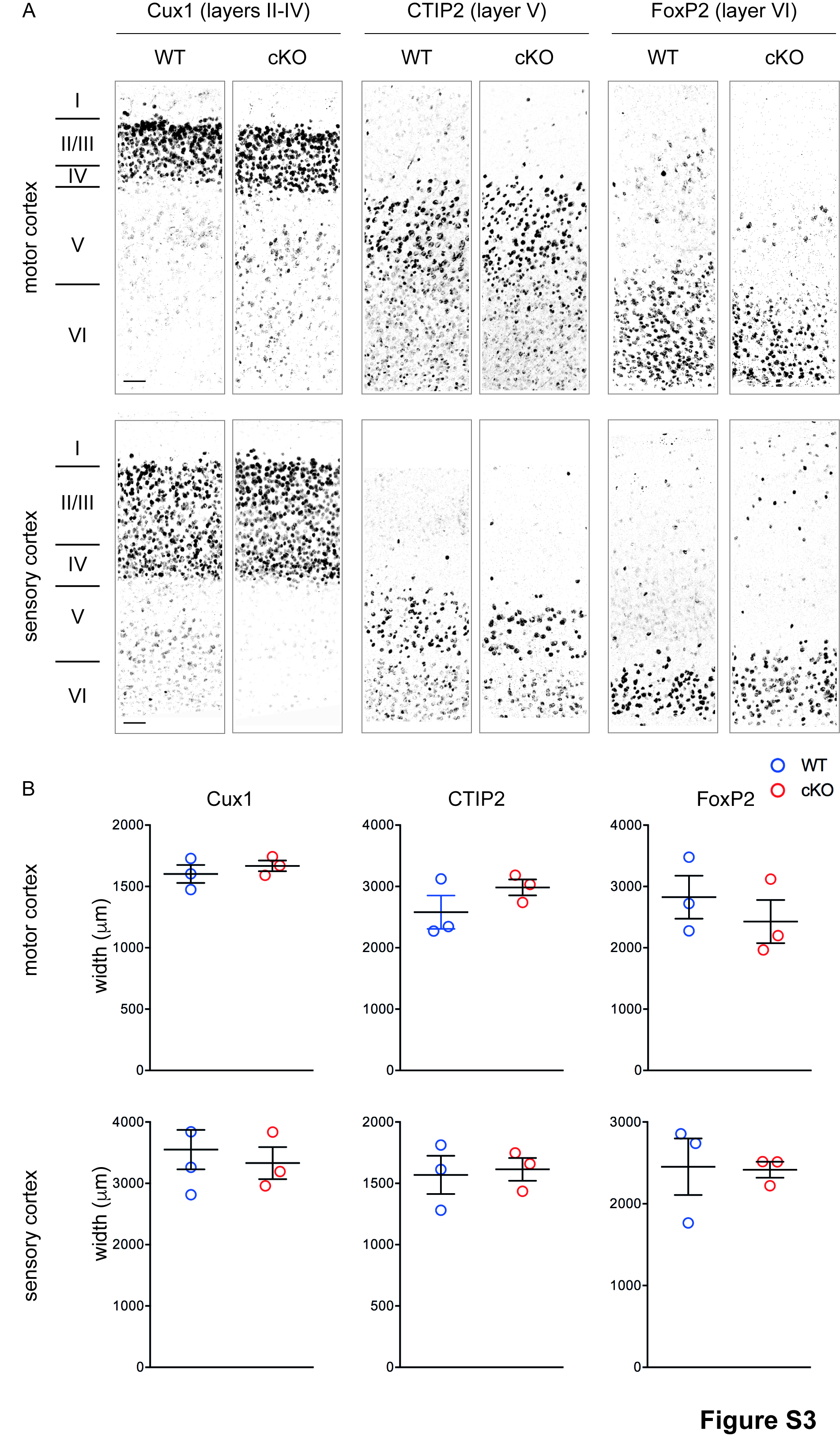

Supplement: Supplementary file 4 — Supplementary Figure 3 [file 41420_2022_854_MOESM4_ESM.tif]

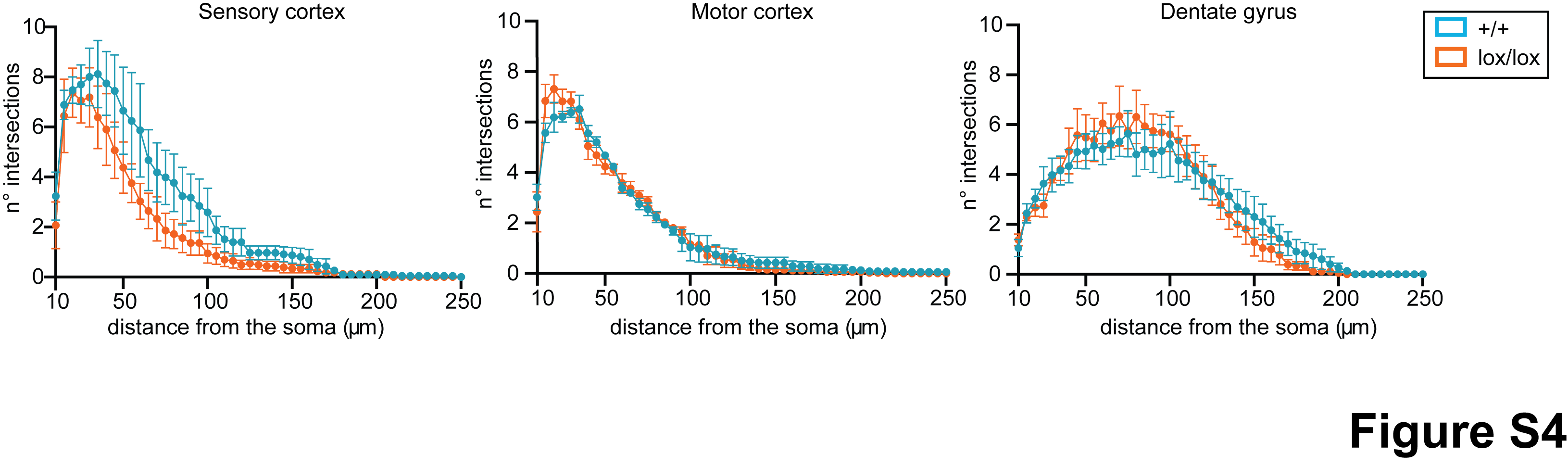

Supplement: Supplementary file 5 — Supplementary Figure 4 [file 41420_2022_854_MOESM5_ESM.tif]

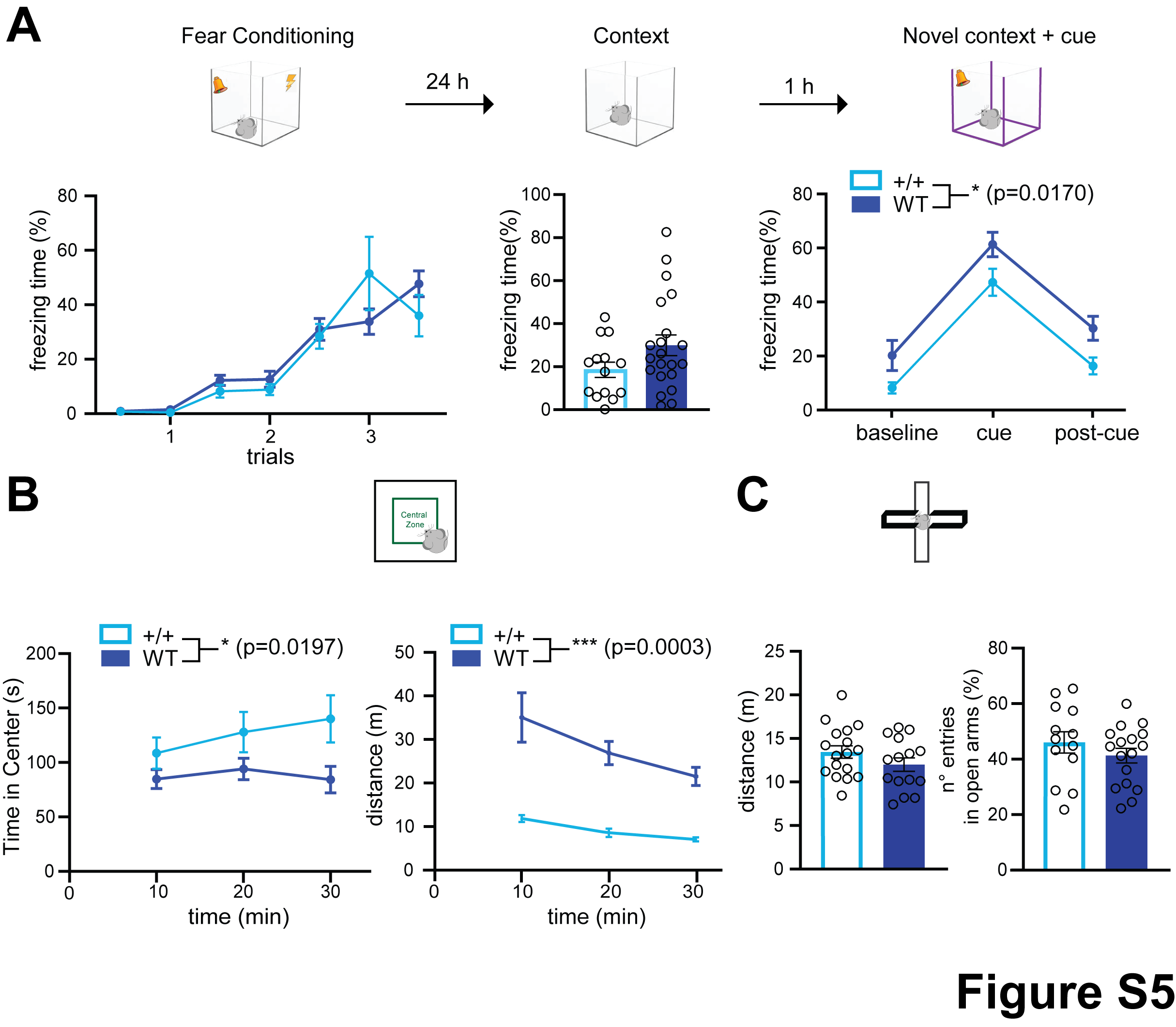

Supplement: Supplementary file 6 — Supplementary Figure 5 [file 41420_2022_854_MOESM6_ESM.tif]

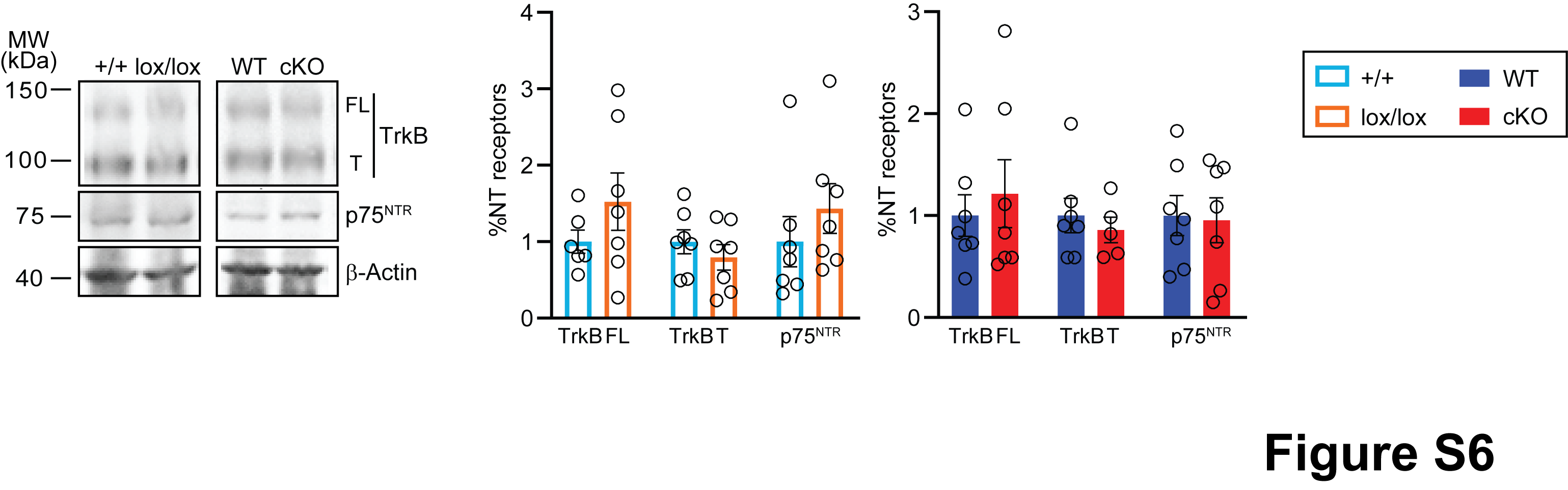

Supplement: Supplementary file 7 — Supplementary Figure 6 [file 41420_2022_854_MOESM7_ESM.tif]

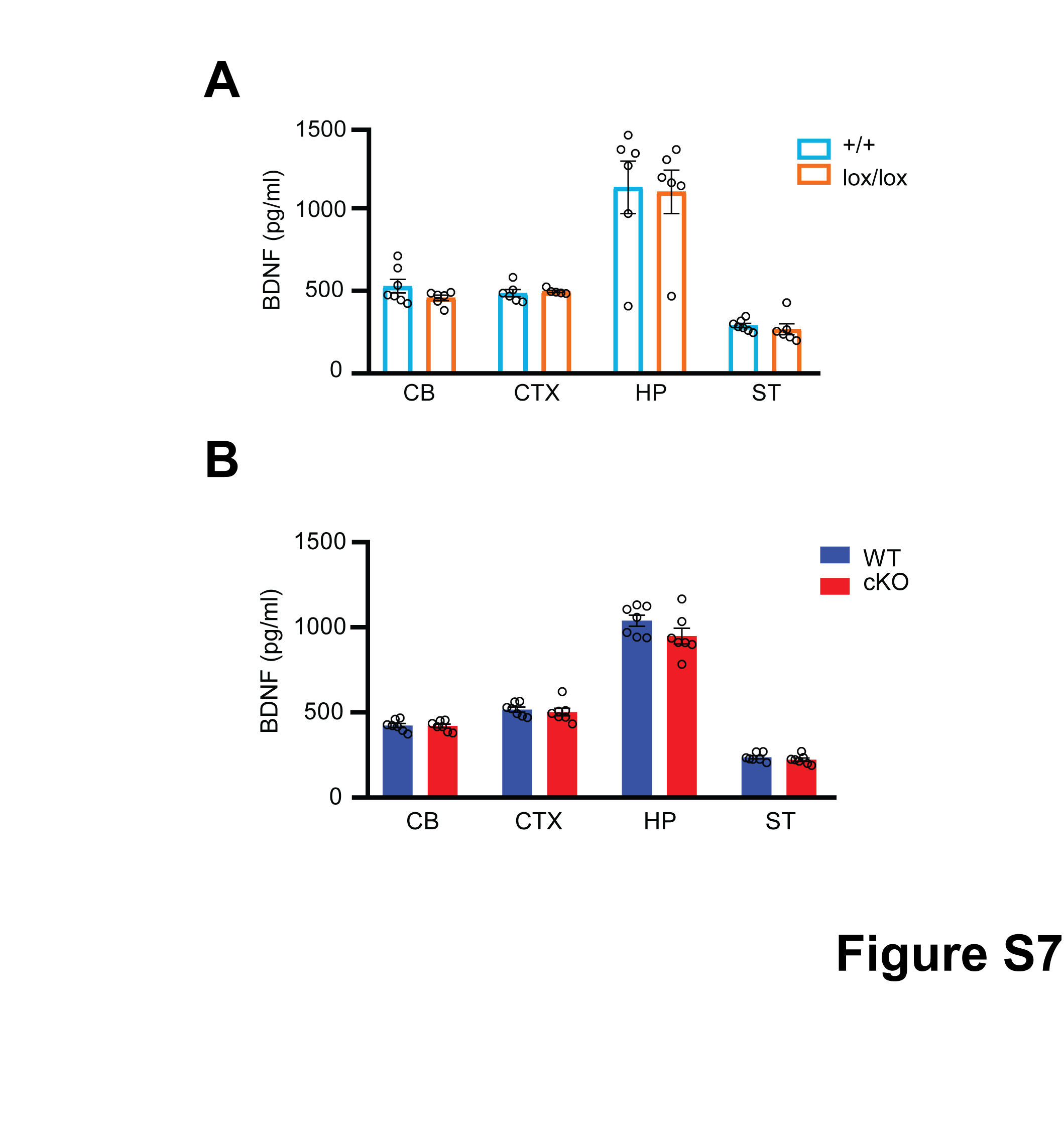

Supplement: Supplementary file 8 — Supplementary Figure 7 [file 41420_2022_854_MOESM8_ESM.tif]
